# S100 proteins in IgA vasculitis and other systemic vasculitides – from pathogenic mechanisms to clinical biomarkers: a systematic review

**DOI:** 10.3389/fimmu.2026.1802262

**Published:** 2026-03-25

**Authors:** Zofia Podraza, Emilia Złotnik-Szwech, Małgorzata Mizerska-Wasiak

**Affiliations:** Department of Pediatrics and Nephrology, Medical University of Warsaw, Warsaw, Poland

**Keywords:** calgranulin C, calprotectin, IgA vasculitis, S100 proteins, S100A10, S100A4, vasculitis

## Abstract

**Background:**

IgA vasculitis (IgAV) is a small-vessel vasculitis characterized by immune complex deposition, neutrophil activation, and endothelial injury. S100 proteins are recognized mediators of inflammation and vascular damage; however, their specific biological and clinical relevance in IgAV remains incompletely understood.

**Objectives:**

This systematic review aimed to synthesize current evidence on the mechanistic and clinical roles of S100 proteins in vasculitides, focusing on IgAV, and to evaluate their potential utility as biomarkers of disease activity, organ involvement, and prognosis.

**Methods:**

A PRISMA-compliant systematic search was conducted in PubMed, Embase, Scopus, and Web of Science up to November 18, 2025. Original human studies investigating S100A8/9, S100A12, S100A4, and S100A10 in any form of vasculitis or related vascular inflammation were included. Data were synthesized narratively following an independent risk-of-bias assessment.

**Results:**

Fifty-four studies met the inclusion criteria. Available evidence supports the role of S100A8/9 and S100A12 as markers of neutrophil-driven inflammation and disease activity in systemic vasculitides, with emerging evidence suggesting relevance in IgAV. In contrast, findings on S100A4 and S100A10 were fragmentary and indirect, indicating a mechanistic contribution but lacking sufficient IgAV-specific data.

**Conclusions:**

S100 proteins may act as mediators in the IgAV inflammatory cascade. However, the current evidence base remains fragmented. Although S100A8/9 appears promising, standardized prospective studies are required for S100A12, S100A4, and S100A10 to establish their clinical validity for risk stratification.

## 1 Introduction

Vasculitis is characterized by immune dysregulation and endothelial injury. While S100 proteins are known mediators of inflammation, current evidence regarding their specific pathogenic role in IgA vasculitis (IgAV) remains fragmented and inconclusive. A comprehensive synthesis of current knowledge is therefore needed to clarify how S100-mediated pathways influence vascular inflammation, immune activation, and clinical manifestations in vasculitis.

The objective of this systematic review is to evaluate the biological, mechanistic, and clinical relevance of S100 proteins in vasculitides, with particular emphasis on IgAV. Specifically, the review aims to determine how differences in S100 protein expression or activity relate to key inflammatory mechanisms and clinically relevant outcomes. This review encompasses studies of individuals (both adults and children) with vasculitis or vascular inflammatory injury, as well as *in vitro* studies examining S100-driven inflammatory pathways.

### 1.1 General characteristics of the S100 protein family

The S100 family consists of small proteins that bind calcium through unique EF-hand structures. Most S100 genes are localized on chromosome 1q21, a region central to genomic regulation of tissue development. Within the cell, these proteins act as regulators, managing calcium homeostasis, cytoskeletal organization, and signal transduction through the modulation of various enzymes and protein kinases. This intracellular activity is crucial for cellular growth, energy metabolism, and apoptosis (1). Under conditions of physiological stress or tissue damage, S100 proteins are secreted into the extracellular space, where they act as damage-associated molecular patterns (DAMPs). By binding to receptors such as the receptor for advanced glycation end-products (RAGE) and Toll-like receptor 4 (TLR4), they bridge the gap between innate and adaptive immunity, often amplifying local inflammatory cascades. Specific members, notably S100A8, S100A9, and S100A12, function as chemoattractants that drive leukocyte infiltration to injury sites. Additionally, some isoforms contribute to host defense by sequestering essential transition metals (zinc and magnesium) via chelation. Given their high expression in the skin, S100 proteins are uniquely positioned to influence the localized vascular and epithelial inflammation typical of IgAV, making them significant candidates for both diagnostic biomarkers and targeted therapies (2).

### 1.2 Pathogenesis of IgA vasculitis

The pathogenesis of IgAV involves a multistep process progressing from immune dysregulation to small-vessel injury. Initially, respiratory or gastrointestinal infections trigger Toll-like signaling and cytokine release. These mechanisms drive excessive B-cell activation and increased IgA1 production in genetically susceptible individuals (3, 4). S100 proteins, such as S100A8/A9 (calprotectin) and S100A12 (calgranulin C), activate Toll-like receptors (TLRs), especially TLR4, amplifying innate immune signaling and early immune activation. This immune dysregulation impacts B-cell glycosyltransferases. Consequently, aberrant O-glycosylation of the IgA1 hinge region results in galactose-deficient IgA1 (Gd-IgA1). This process is driven by reduced activity of C1GALT1 (Core 1 β1,3-galactosyltransferase 1) and its molecular chaperone *COSMC* (C1GALT1-specific chaperone 1), or by enhanced function of sialyltransferases, such as ST6GalNAc-II (α-N-acetylgalactosaminide α-2,6-sialyltransferase 2) (5, 6).

Exposed GalNAc (N-acetylgalactosamine) determinants on Gd-IgA1 serve as neoepitopes, inducing anti-Gd-IgA1 autoantibodies production and promoting the formation of circulating immune complexes (ICs). These ICs deposit within the vascular endothelium of multiple organs (4). During immune activation, increased expression of TfR1 (Transferrin Receptor 1, also known as CD71) on mesangial cells promotes the deposition of Gd-IgA1-containing immune complexes, thereby triggering mesangial activation and glomerular injury (3, 7).

The deposition of ICs triggers complement activation, primarily through the alternative and lectin pathways. Amplification through the alternative pathway (factors B and D) enhances C3 and C5 convertase formation, generating C3b, C5a, and the membrane attack complex (MAC, C5b-9), all of which contribute to leukocyte recruitment and endothelial injury (8, 9). In both renal and cutaneous lesions, complexes containing mannose-binding lectin (MBL), MBL-associated serine proteases 1 and 2 (MASP-1/2), and C4d — components characteristic of the lectin pathway — are typically detected. In contrast, C1q, a key element of the classical pathway, is rarely observed (10–12).

**Figure 1:**
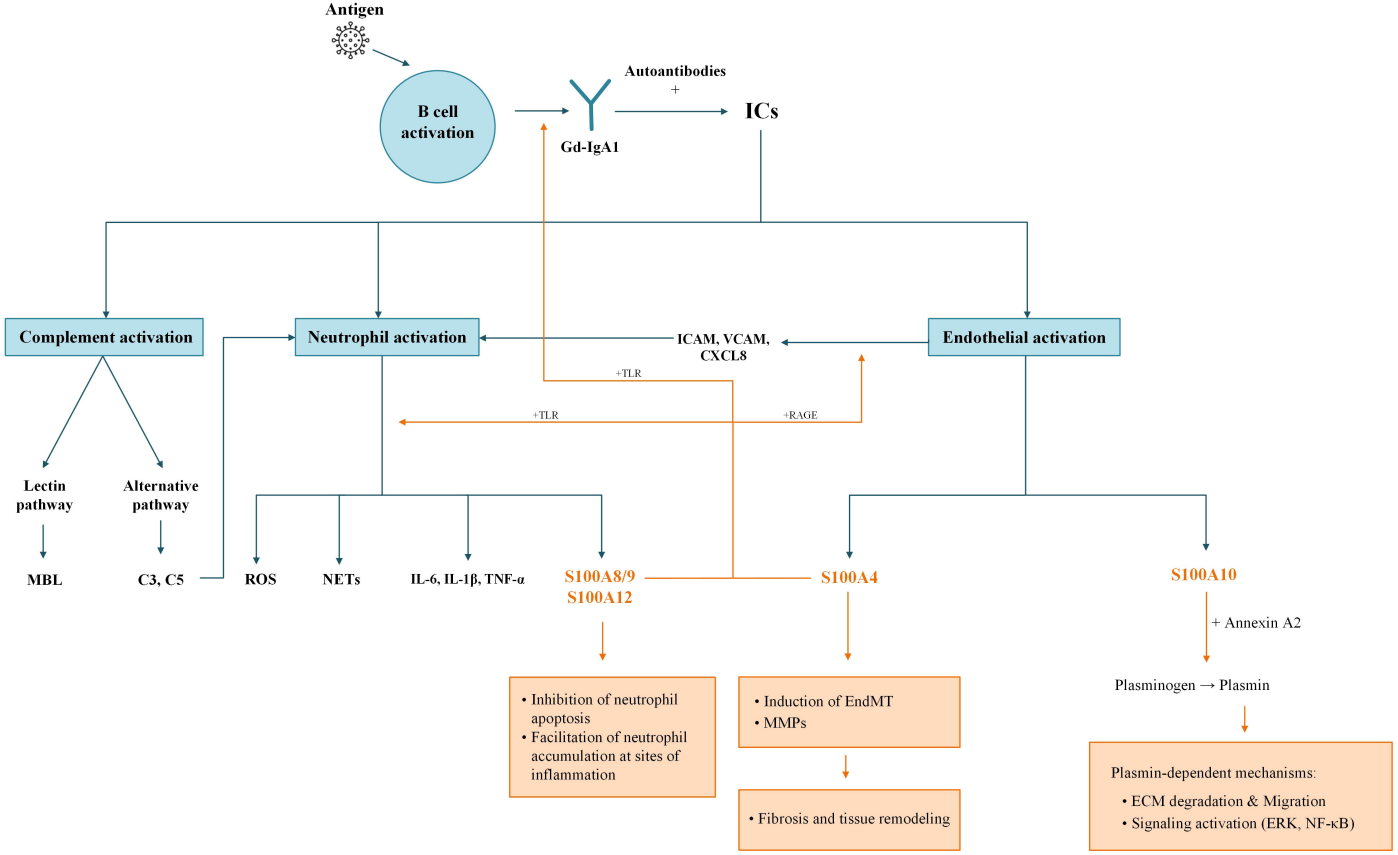
Proposed pathogenic mechanisms of S100 proteins in IgAV. The figure illustrates the pathogenesis of IgAV and the specific roles of various S100 proteins. The process begins with B-cell activation and the production of Gd-IgA1, which forms ICs. ICs trigger complement, neutrophil and endothelial activation. Activated neutrophils secrete S100A8/9 and S100A12, while activated endothelium releases S100A4 and S100A10. S100A8/9, S100A12, and S100A4 establish a pro-inflammatory feedback loop by stimulating B cells via TLR to increase immunoglobulin production and also activate neutrophils to release ROS, NETs, and cytokines (IL-6, IL-1β, TNF-α). Furthermore, S100A8/9 and S100A12 inhibit neutrophil apoptosis and facilitate their accumulation at inflammatory sites. S100A4 promotes fibrosis and tissue remodeling by inducing EndMT and stimulating MMP secretion. Finally, S100A10 interacts with Annexin A2 to convert plasminogen into plasmin, triggering plasmin-dependent mechanisms such as ECM degradation and signaling activation (ERK, NF-κB). Blue arrows – pathogenesis of IgAV, orange arrows – S100 proteins mechanisms. C3, C5, complement components 3 and 5; ECM, extracellular matrix; EndMT, endothelial-to-mesenchymal transition; ERK, extracellular signal-regulated kinase; Gd-IgA1, galactose-deficient IgA1; ICAM, intercellular adhesion molecule; ICs, immune complexes; IL, interleukin; MAPK, mitogen-activated protein kinase; MBL, mannose-binding lectin; MMPs, matrix metalloproteinases; NETs, neutrophil extracellular traps; NF-κB, nuclear factor kappa-light-chain-enhancer of activated B cells; RAGE, receptor for advanced glycation end products; ROS, reactive oxygen species; TLR, Toll-like receptor; TNF-α, tumor necrosis factor-alpha; VCAM, vascular cell adhesion molecule.

C5a and IgA engagement of FcαRI (IgA Fc receptor, CD89), expressed on neutrophils and monocytes, activates these cells, inducing degranulation, reactive oxygen species (ROS) production, and neutrophil extracellular trap (NET) formation, which contribute to vascular damage (13–15). Activated endothelial cells express adhesion molecules, including E-selectin, ICAM-1 (Intercellular Adhesion Molecule 1), and VCAM-1 (Vascular Cell Adhesion Molecule 1), enabling neutrophil rolling, adhesion, and transmigration (16–18). Released proteases, such as elastase and MMP-9 (Matrix Metalloproteinase-9), and ROS degrade basement membrane and junctional proteins, increasing vascular permeability and causing hemorrhagic lesions typical of IgAV (15, 18). Furthermore, released S100 proteins act as damage-associated molecular patterns (DAMPs), amplifying the inflammatory cycle and actively shaping the immune response (**Figure 1**).

Collectively, this cascade of immune dysregulation, complement activation, and neutrophil-mediated endothelial damage results in clinical manifestations.

## 2 Materials and methods

### 2.1 Protocol and registration

This systematic review was conducted in accordance with the Preferred Reporting Items for Systematic Reviews and Meta-Analyses (PRISMA) 2020 guidelines, and the PRISMA checklist is provided in the Supplementary Materials (**Supplementary Table 1**) (19). The protocol for this review was not preregistered in PROSPERO or any other prospective register.

### 2.2 Eligibility criteria

Studies were selected based on the following criteria:

- Inclusion criteria: (1) Original research articles; (2) Studies investigating S100 proteins (particularly S100A8/A9, S100A12, S100A4, and S100A10) in the context of vasculitis or diseases involving vascular inflammation or injury (e.g., IgAV, ANCA-associated vasculitis (AAV), lupus nephritis (LN), atherosclerosis, acute kidney injury); (3) Studies utilizing human biological samples or human-derived cell lines.
- Exclusion criteria: (1) Conference abstracts, case reports, editorials, commentaries, and review articles; (2) Studies focusing on oncology, metabolic, or neurological diseases that lacked transferable mechanistic relevance to vascular inflammation; (3) Animal studies or *in vitro* studies conducted on non-human cell lines; (4) Duplicate or incomplete datasets.

While review articles were excluded from the systematic data synthesis and risk of bias assessment, selected reviews were used to provide a mechanistic context and theoretical background, as well as mechanical contexts for the introduction of protein subgroups.

### 2.3 Information sources and search strategy

A comprehensive literature search was conducted in PubMed, Embase, Scopus, and Web of Science to identify relevant studies from inception to November 18, 2025. The search strategy combined Medical Subject Headings (MeSH) with free-text keywords applied to Title/Abstract fields to balance sensitivity and specificity. Separate search strings were constructed for each protein subgroup. An example search string for S100A8/A9 was: “S100A8/9” OR “S100A8” OR “S100 A8” OR “S100A9” OR “S100 A9” OR “calprotectin” OR “calgranulin A” OR “calgranulin B” OR “MRP8” OR “MRP14” OR “MRP8/14” AND “IgA vasculitis” OR IgAV OR “Henoch-Schönlein purpura” OR “Henoch Schoenlein purpura” OR HSP OR “IgA-associated vasculitis” OR “Immunoglobulin A vasculitis” OR “IgA mediated vasculitis” OR “small vessel vasculitis” OR “leukocytoclastic vasculitis” OR LCV OR “cryoglobulinemic vasculitis” OR “cryoglobulinemia” OR “immune complex vasculitis” OR “hypersensitivity vasculitis” OR “systemic vasculitis”. Complete search strings for all databases are provided in **Supplementary Table 2**. Grey literature sources were not searched, as the focus of this review was strictly on peer-reviewed mechanistic and clinical studies providing detailed biological insights. No language or time restrictions were applied.

### 2.4 Selection procedure

Study selection was performed independently by two reviewers (Z.P. and E.Z.-Sz.). Zotero reference management software (version 7.0.27; Corporation for Digital Scholarship, Vienna, VA, USA, 2024) was utilized for data handling. Any disagreements were resolved through discussion, with a third reviewer serving as an arbitrator when consensus could not be reached.

### 2.5 Data extraction and management

One reviewer (Z.P.) extracted data using a standardized data extraction form, and a second reviewer (E. Z.-Sz.) independently verified all extracted information. The following variables were collected: publication year, country/setting, study type/model/population, sample size, S100 protein investigated, study aim, exposure or intervention, comparator, outcomes assessed, and key findings (**Supplementary Table 3**).

### 2.6 Synthesis methods

Data synthesis was organized by individual S100 proteins and structured into two distinct sections. The first section consists of the mechanistic functions and pathogenic role of the protein at the molecular level. The second section focuses on clinical evidence, specifically evaluating correlations with disease activity and the utility of the protein as a diagnostic biomarker in systemic vasculitides. This structured narrative approach enabled consistent comparison across S100 proteins and diseases despite substantial heterogeneity in study designs, populations, biomarkers, and outcomes. No meta-analysis was performed due to this heterogeneity.

### 2.7 Assessment of risk of bias

Risk of bias was assessed by two independent reviewers using validated tools tailored to each study design. The following assessment tools were used: Newcastle-Ottawa Quality Assessment Form for Case-Control Studies (20); the JBI Critical Appraisal Checklist for Analytical Cross-Sectional Studies (21); QUIPS (Quality In Prognosis Studies) (22); the NIH Quality Assessment Tool for Before-After (Pre-Post) Studies with No Control Group (23), JBI Critical Appraisal Checklist for Randomized Controlled Trials (24), and OHAT Risk of Bias Rating Tool for Human and Animal Studies (25).

The certainty of evidence was evaluated using a Grading of Recommendations Assessment, Development, and Evaluation (GRADE) tool (26).

## 3 Results

### 3.1 Study selection and characteristics

**Figure 2:**
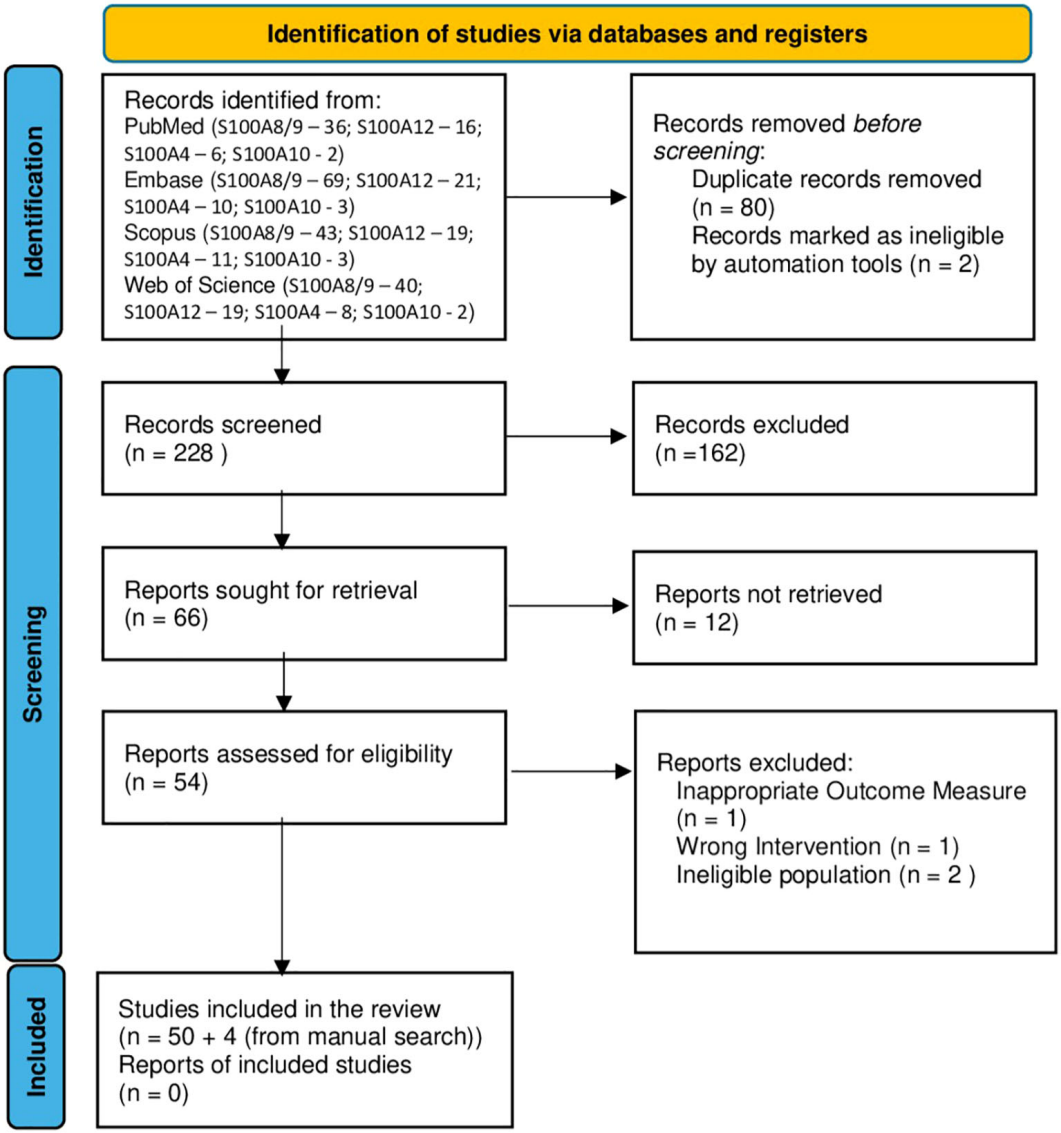
PRISMA 2020 flow diagram (19).

Following the removal of duplicates, 228 records were identified (for S100A8/9: 167, S100A12: 62, S100A4: 20, S100A10: 3, with overlaps between categories). Titles and abstracts were screened, and irrelevant records were excluded. A total of 67 full-text articles were assessed for eligibility. Of these, 15 articles were excluded (5 due to failing inclusion criteria, and 12 articles were not retrieved). Additionally, 4 relevant studies were identified through manual reference screening of included papers and reviews. Finally, 54 studies met the inclusion criteria and were included in the qualitative synthesis. The study selection process is presented in the PRISMA flow diagram (**Figure 2**) (19).

### 3.2 Risk of bias assessment

The risk of bias assessment for included studies is detailed in the Supplementary Materials (**Supplementary Table 4**). Given the heterogeneity of study designs, multiple assessment tools were employed. Overall, the majority of clinical studies demonstrated moderate to high methodological quality. The most frequent sources of potential bias were small sample sizes and the lack of blinding in outcome assessment.

### 3.3 Certainty of evidence assessment

The certainty of evidence was evaluated using a GRADE tool (26). The assessment covered all standard domains, including risk of bias, inconsistency, indirectness, imprecision, and publication bias. Observational clinical studies were initially rated as low certainty, whereas purely mechanistic or in silico data were considered very low by default. Certainty was upgraded for consistent findings across independent cohorts or robust experimental validation. Evidence was downgraded when serious or very serious concerns were identified in any domain.

### 3.4 Protein S100A8/9

#### 3.4.1 Molecular functions of S100A8/S100A9 complex

Released during neutrophil activation, the calprotectin complex (S100A8/9) serves as both a marker and modulator of inflammation (27). While intracellularly it regulates signaling pathways controlling inflammatory responses, its extracellular release triggers its function as a key damage-associated molecular pattern (DAMP), amplifying local inflammation and promoting leukocyte recruitment (27).

Extracellular calprotectin primarily interacts with TLR4 and the receptor for advanced glycation end products (RAGE) (28). Activation of TLR4 by the S100A8/9 complex initiates a signaling cascade involving MyD88 and NF-κB, leading to upregulated transcription of pro-inflammatory cytokines such as tumor necrosis factor-alpha (TNF-α) and interleukins (IL-6, IL-8, and IL-23) (29). Significantly, simultaneous blockade of TLR4 and RAGE *in vitro* abolishes the pro-inflammatory effects of S100A8/9, underscoring the essential contribution of these receptors to calprotectin signaling (30).

Concurrently, RAGE-dependent MAPK signaling (p38, JNK, ERK1/2) enhances the production of chemokines (e.g., CXCL8, monocyte chemoattractant protein-1 - MCP-1) and the expression of endothelial adhesion molecules, such as ICAM-1 and VCAM-1. This promotes neutrophil recruitment and leukocyte transmigration into inflamed vascular tissue—a mechanism consistent with the inflammatory cell infiltration characteristic of IgAV. It has been observed that the induction of ICAM-1, VCAM-1, and IL-6 required prior endothelial activation, whereas MCP-1 production increased independently of such priming. These findings suggest that distinct calprotectin-driven signaling pathways may be engaged depending on the activation state of target cells, potentially explaining the heterogeneous vascular changes observed in IgAV (31, 32).

Beyond its effects on endothelial cells, the S100A8/9 complex directly modulates neutrophil functions. S100A8/9 has been shown to prolong neutrophil survival by inhibiting apoptosis, facilitating their accumulation at sites of inflammation (33).

In addition to its role in innate immunity, calprotectin also significantly influences adaptive immune responses. It participates in CD8+ T-cell priming by antigen-presenting cells and serves as a co-stimulatory mediator in conjunction with CD40/CD40L signaling, potentially promoting T-cell tolerance breakdown (33).

Calprotectin demonstrates a dual nature, functioning also as a regulator within the intracellular environment. Intracellularly, calprotectin can mediate anti-inflammatory effects by suppressing NF-κB activation and downregulating the expression of IL-1β, IL-6, and TNF-α (31). Furthermore, Bai et al. (30) demonstrated that S100A8/9 limits myeloperoxidase-antineutrophil cytoplasmic antibody (MPO-ANCA)–induced ROS generation in neutrophils and reduces oxidative stress–dependent NET formation. S100A8 also participates in the negative regulation of leukocyte adhesion and transmigration by diminishing p38 MAPK phosphorylation (34). This dual functionality suggests a complex homeostatic role, where intracellular S100 proteins may limit oxidative stress, whereas their massive extracellular release during degranulation overrides these checkpoints, driving the dysregulated inflammation and endothelial damage typical of IgAV.

Therefore, calprotectin links innate and adaptive immunity, integrating neutrophil activation, endothelial injury, and T-cell regulation. It is involved in both pro-inflammatory and regulatory mechanisms, making it a promising biomarker of disease activity and a potential target for future therapeutic strategies in IgAV.

#### 3.4.2 Clinical relevance of S100A8/9

Expression of S100A8/9 is strongly upregulated across a wide spectrum of inflammatory diseases. In recent years, its utility as a biomarker of disease activity has been characterized in various forms of vasculitis. In ANCA-associated vasculitis (AAV), elevated serum and urinary S100A8/9 levels correlate with disease activity and decline following effective therapy (sample sizes ranging from n=42 to n=169) (30, 35–37). Importantly, patients who experienced relapses during follow-up demonstrated a smaller reduction in serum S100A8/9, and higher levels were associated with an increased risk of renal involvement (38). Romand et al. (39) in a cohort of 76 AAV patients found that increased serum S100A8/9 at month 6 predicted renal function decline in renal function over the following 12 months. These findings suggest that S100A8/9 may serve not only as a marker of current activity but also as an indicator of disease progression—an observation that could have relevance for IgAV, where relapses and renal involvement are key determinants of clinical severity.

In Kawasaki disease (KD), S100A8/9 levels also correlate with inflammatory activity (40, 41). Fecal S100A8/9 has additionally been identified as a marker of coronary artery injury (42–44). Serum S100A8/9 typically decreases after IVIG therapy, whereas persistently elevated concentrations have been associated with aneurysm formation in a cohort of 46 KD patients (41). In contrast, S100A8/9 levels remain unchanged following infliximab (anti-TNF) administration, despite the normalization of other inflammatory markers such as CRP (n=11 infliximab-treated patients) (45). The fact that S100A8/9 levels decrease after IVIG but persist after anti-TNF treatment suggests that infliximab may successfully reduce systemic cytokine-mediated symptoms (e.g., fever) but fails to suppress the local, neutrophil-driven vascular inflammation completely. Clinically, this implies that S100A8/9 offers superior sensitivity in detecting residual vascular inflammation compared to conventional markers, particularly in patients undergoing biological therapy.

Fecal S100A8/9 is an established marker of gastrointestinal involvement in Behçet’s disease (BD) and may facilitate the detection of subclinical intestinal disease in otherwise asymptomatic patients (n=30) (46, 47). Levels are significantly higher in individuals with intestinal ulcers compared with those who have normal colonoscopy findings (n=44 BD patients) (48, 49). Urinary S100A8/9 has also been proposed as a potential marker of disease activity in Behçet’s disease (50).

Findings in giant cell arteritis (GCA) remain inconsistent. Saut et al. (n=22) (51) reported higher levels in active disease vs. remission, though levels did not predict relapse. However, several studies (from n=41 to n=123) have found no meaningful association between S100A8/9 levels and disease activity in GCA, although its levels tend to be elevated compared with those of healthy controls (52–54). In Takayasu arteritis (TA), serum S100A8/9 correlates with both clinical and angiographic activity, independent of the corticosteroid dose (n=85) (52).

S100A8/9 has also demonstrated value in autoimmune diseases in which vascular involvement is common. In systemic lupus erythematosus (SLE), S100A8 concentrations in serum, urine, and saliva have been shown to correlate with disease activity (n=249) (55). Also, in the study by Homa-Mlak et al. (n=59) (28), serum S100A8/9 levels >2.37 µg/mL were associated with a high probability of a moderate or severe course of SLE. Among patients with rheumatoid arthritis (RA), elevated S100A8/9 levels are associated with increased vascular risk and cumulative organ damage (56). In a study by Zhao et al. (57), serum calprotectin was significantly elevated in patients with antiphospholipid antibodies, suggesting its potential as a marker of microvascular manifestations, such as Antiphospholipid Syndrome nephropathy and livedo reticularis.

S100A8/9 measurable presence in serum, urine, and stool highlights its wide-ranging clinical utility and underscores its complex role in systemic inflammation. Collectively, the available data support the conclusion that calprotectin serves as a sensitive marker of inflammatory activity in small- and medium-vessel vasculitides, likely reflecting the distinct, neutrophil-driven pathogenesis of small-vessel vasculitides compared to the macrophage/T-cell dominance in large-vessel diseases.

#### 3.4.3 S100A8/9 in IgAV

In IgAV, findings regarding S100A8/9 remain inconsistent. The available data regarding S100A8/9 concentrations exhibit significant variability across the literature, yet several studies have demonstrated an association between S100A8/9 levels and clinical disease activity. Srsen et al. (n=69) (58) observed that higher serum S100A8/9 concentrations correlated with the extent of cutaneous involvement. However, findings regarding fecal S100A8/9 and gastrointestinal manifestations remain likely inconsistent, reflecting the clinical heterogeneity of IgAV and methodological variability in assessing inflammatory activity (59–61). Kanik et al. (n=66) (62) suggested that fecal S100A8/9 may serve as a marker of risk for both gastropathy and nephropathy in children, while Paek et al. (n=69) (61) demonstrated significantly higher levels in patients with lower gastrointestinal tract involvement.

Regarding tissue expression, Kung et al. (n=21) (63) revealed a paradoxically increased S100A8/9 expression in skin biopsies from patients with skin-limited IgAV compared to those who developed nephropathy. These findings suggest that S100A8/9 levels in skin tissue may mirror localized inflammatory activity characteristic of the cutaneous phenotype. Consequently, differential expression patterns could hold clinical relevance, potentially serving as an indicator to distinguish between skin-limited and systemic disease phenotypes.

#### 3.4.4 S100A8/9 and renal involvement

Renal involvement is a major determinant of prognosis in both IgAV and AAV. Anton-Pampols et al. (n=138) (64) proposed that elevated serum S100A8/9 may serve as an early marker of renal involvement in AAV, potentially preceding clinically apparent manifestations. Similarly, Martínez Valenzuela et al. (65) (n=42 AAV patients) found higher serum S100A8/9 concentrations in AAV patients with active nephritis (proteinuria, hematuria) and a correlation with decreased glomerular filtration rate GFR. In contrast, studies focusing on stable chronic kidney disease (CKD) have yielded different results. Seibert et al. (66) (n=143) did not confirm a correlation between urinary S100A8/9 and GFR decline across various primary inflammatory kidney diseases. Consistent with these findings, Seibert et al. (67) demonstrated no significant differences in urinary S100A8/9 between patients with primarily inflammatory versus non-inflammatory stable CKD. These findings suggest that the diagnostic value of S100A8/9 may depend on the underlying disease mechanism (active vasculitis vs. chronic damage) and the phase of the disease.

Comparable observations have been made in IgAV. Patients with more advanced renal lesions (ISKDC-International Study of Kidney Disease in Children grades III–V) (n=30) exhibited significantly higher serum S100A8/9 levels, which also correlated with increased E-selectin concentrations, indicating underlying endothelial dysfunction (68). However, fecal S100A8/9 levels in IgAV (n=47) were not associated with renal damage parameters such as proteinuria or hematuria, suggesting that fecal S100A8/9 reflects localized gastrointestinal inflammation rather than renal pathology (60).

#### 3.4.5 Certainty of evidence for S100A8/9

Certainty of evidence using GRADE assessment for S100A8/9 in systemic vasculitides (AAV, KD) was evaluated as moderate due to consistent findings across large cohorts. For IgAV, the certainty remains low due to smaller clinical samples and higher heterogeneity in reported outcomes compared to other systemic vasculitides.

### 3.5 Protein S100A12

#### 3.5.1 Molecular functions of S100A12

S100A12 (calgranulin C or EN-RAGE) is a pro-inflammatory cytokine predominantly secreted by neutrophils and, additionally, at lower concentrations, by monocytes and macrophages (69). Under inflammatory conditions, S100A12 is released as a DAMP molecule capable of binding to receptors such as RAGE and TLR4. This binding activates NF-κB signaling, induces the production of pro-inflammatory cytokines, and promotes ROS generation, mirroring the activity of calprotectin (70). Through these mechanisms, S100A12 amplifies innate immune responses and contributes to vascular remodeling in the course of vasculitis (69, 71).

The activity of S100A12 is naturally regulated by the soluble form of RAGE (sRAGE). sRAGE acts as a “decoy receptor” that binds to circulating S100A12, thereby preventing it from interacting with membrane-bound RAGE and blocking the downstream inflammatory cascade. Therefore, lower levels of sRAGE may exacerbate vascular inflammation due to insufficient neutralization of S100A12.

Circulating S100A12 (and other S100 proteins) binds to RAGE receptor and promotes vascular inflammation, suggesting that S100A12 may directly participate in vascular pathology rather than serving solely as a passive marker of inflammation (72).

Although S100A12 expression strongly correlates with S100A8/9, its circulating concentration is at least tenfold lower, and its biological activity is comparatively weaker. Nevertheless, S100A12 levels correlate tightly with disease activity across multiple inflammatory conditions, supporting its utility as a disease activity biomarker in vasculitides, RA, inflammatory bowel disease, and psoriasis (73).

Correlation analyses have shown that S100A12 is positively associated with neutrophils and dendritic cells, and negatively associated with activated CD8+ T cells, B cells, and Th2 cells (74). These findings indicate that S100A12 is predominantly involved in the innate immune phase, rather than adaptive immunity, reinforcing its relevance to early inflammatory events such as vascular injury.

#### 3.5.2 Clinical relevance of S100A12

In a study of n=18 patients, a serum S100A12 concentration of 24 ng/mL was proposed as the optimal cut-off for identifying active vasculitis, with a sensitivity of 89% and specificity of 67% (75). Chen et al. (n=138) (56) reported correlations between S100A12 and traditional vascular risk factors—diabetes, dyslipidemia, and overweight—as well as systemic inflammation and organ damage.

In KD, numerous studies show that S100A12 is markedly elevated during the acute phase (from n=75 to n=174) (43, 76–78). Bioinformatic analyses consistently identify S100A12 gene upregulation as a key signature of acute KD (76–79). Its serum levels decrease after treatment with intravenous immunoglobulin (IVIG) and methotrexate (n=46, n=153, respectively) (41, 80). But not after infliximab (n=43) (45). This observation is the same as the one described above for S100A8/9, pointing to persistent local neutrophil-driven inflammation despite biological therapy. Consequently, S100A12 also demonstrates superior sensitivity over conventional markers in detecting residual vascular inflammation. Furthermore, in treatment-nonresponsive patients, S100A12 levels rise again after an initial decline, indicating persistent neutrophil activation as a potential mechanism of treatment resistance (45).

Crucially, S100A12 expression on endothelial cells remains elevated for prolonged periods in KD patients (n=50) who develop coronary artery aneurysms, suggesting that sustained activation of S100A12-dependent pathways may drive the progression of vascular injury (81). S100A12 also stimulates monocytes to produce IL-8, enhancing neutrophil recruitment and worsening endothelial dysfunction in KD (n=30) (82). These findings indicate that S100A12 not only reflects inflammation but also actively contributes to vascular damage and remodeling.

Similarly, S100A12 plays a substantial role in AAV (30, 36). Serum levels correlate with the Vasculitis Damage Index (VDI), Birmingham Vasculitis Activity Score (BVAS), leukocyte count, CRP, creatinine, and other markers of disease activity (n=79, n=56) (35, 83). Regarding the protective role of sRAGE, Yoon et al. (n=75) (84) observed that baseline EN-RAGE ≤84.37 ng/mL and sRAGE ≥1.82 ng/mL were associated with improved survival. Notably, in multivariable analysis, elevated sRAGE remained an independent predictor of lower mortality, highlighting the importance of the ligand-decoy receptor balance in AAV prognosis.

S100A12 has also been studied in other vascular diseases. In Takayasu arteritis, increased fecal S100A12 levels were observed in patients (n=30) with gastrointestinal symptoms, suggesting its ability to reflect local vascular inflammation within the gastrointestinal tract (85). A similar mechanism may apply to IgAV, where abdominal symptoms arise from small-vessel involvement of the mesentery. Elevated S100A12 has also been reported in other biological fluids—for instance, in the urine of patients with active SLE (n=64), supporting its role as a marker of organ-specific inflammation (86). In CKD, serum S100A12 concentrations predict both all-cause and cardiovascular mortality, further linking this protein to severe vascular outcomes (72). However, to date, no original studies have specifically evaluated S100A12 directly in IgAV patients. Its classification as a promising biomarker remains a plausible hypothesis based on mechanistic parallels with other neutrophil-driven vasculitides rather than a confirmed clinical finding.

#### 3.5.3 Certainty of evidence for S100A12

Certainty of evidence using GRADE assessment for S100A12 in AAV and KD was evaluated as moderate, supported by robust experimental validation via RT-qPCR and established mechanistic roles in IL-1β-driven endothelial inflammation. For IgAV, the certainty was evaluated as very low due to severe indirectness and imprecision, as evidence is currently extrapolated from other systemic vasculitides without direct clinical or experimental confirmation in IgAV-specific cohorts.

### 3.6 Protein S100A4

#### 3.6.1 Molecular functions of S100A4

S100A4 (metastasin or fibroblast-specific protein – FSP-1) is a calcium-binding member of the S100 protein family. S100A4 is released into the extracellular space by fibroblasts, activated macrophages, and endothelial cells. Under physiological conditions, its expression is minimal, but it is strongly upregulated in response to cellular stress, inflammation, or tissue injury (87).

Experimental studies have shown that once released extracellularly, S100A4 acts as a DAMP molecule, activating both TLR4 and RAGE. This leads to the activation of NF-κB and MAPK cascades and results in increased expression of cytokines (IL-6, TNF-α), chemokines (MCP-1, CXCL8), and adhesion molecules (ICAM-1, VCAM-1). The downstream effects include endothelial activation and leukocyte recruitment—mechanisms shared with other S100 proteins and also characteristic of IgAV (88).

Furthermore, S100A4 induces the expression of matrix metalloproteinases (MMPs) and promotes epithelial-to-mesenchymal transition (EMT) and endothelial-to-mesenchymal transition (EndMT), driving fibrosis and tissue remodeling. These profibrotic properties may be particularly relevant in the later stages of IgAV, especially in patients with chronic renal involvement and progressive glomerular sclerosis (87).

The contribution of S100A4 to vascular pathology has been demonstrated in immunohistochemical and molecular studies of carotid atherosclerotic plaques, where increased S100A4 expression was observed in vascular smooth muscle cells (VSMCs) and correlated with the severity of vascular remodeling. Activation of VSMCs by S100A4 was associated with enhanced cytokine production, extracellular matrix degradation, and weakening of the vascular wall (89). Although these findings predominantly concern large-vessel disease, the underlying mechanisms—endothelial activation, barrier dysfunction, local fibrosis—are conceptually applicable to small-vessel inflammation observed in IgAV (87). Consequently, molecular investigations specifically targeting S100A4 in microvascular inflammation are warranted to verify whether these remodeling mechanisms are conserved across vessels of different calibers. The proposed link between S100A4-driven EndMT and glomerular fibrosis in IgAV is largely speculative and hypothetical, derived from cross-disciplinary evidence in atherosclerosis and oncological models where S100A4 promotes angiogenesis and extracellular matrix degradation.

#### 3.6.2 Clinical relevance of S100A4

Clinical data regarding S100A4 in vasculitides remain limited. In a study involving 126 participants (including 64 with SLE, 53 healthy controls, and 9 with IgAV), Donohue et al. (n=64) (86), S100A4 concentrations were compared across children with SLE, healthy controls, and children with IgAV. No significant differences in S100A4 levels were observed between groups. In contrast, a study by Turnier et al. (n=100) (90) demonstrated that a decrease in urinary S100A4 correlated with clinical improvement in patients with LN. While this study focused on SLE, it highlights the potential utility of S100A4 as a dynamic biomarker of renal inflammatory activity and tissue remodeling.

#### 3.6.3 Certainty of evidence for S100A4

Certainty of evidence using GRADE assessment for S100A4 across all vasculitides, including IgAV, was evaluated as very low due to extreme indirectness and imprecision. Current findings are largely extrapolated from non-vasculitis models, with a critical lack of direct clinical or experimental data in systemic vasculitis cohorts.

### 3.7 Protein S100A10

#### 3.7.1 Molecular functions of S100A10

S100A10 (p11), a member of the S100 protein family, differs from most of its counterparts in that it does not bind calcium ions (Ca^2+^). Despite this, it plays an important role in regulating inflammatory processes and fibrinolysis, primarily through its interactions with membrane-associated proteins and its formation of a complex with annexin A2 (ANXA2). Formation of the ANXA2–S100A10 complex stabilizes S100A10 and enables its localization at the endothelial cell surface, where it participates in local fibrinolytic activity (91).

Functionally, S100A10 mediates the conversion of plasminogen to plasmin. This activity leads to the degradation of extracellular matrix components, resulting in vascular barrier dysfunction and promoting the migration of inflammatory cells into tissues (91). The presence of the ANXA2–S100A10 complex on endothelial cells and macrophages promotes localized fibrinolysis and vascular wall remodeling. Under chronic inflammatory conditions, these mechanisms may contribute to microvascular injury and the formation of necrotic foci—features characteristic of vascular lesions in IgAV (92).

Activation of the S100A10-containing complex further stimulates ERK1/2, PI3K/Akt, and NF-κB signaling pathways, which regulate cell proliferation, adhesion, and survival in inflammatory environments (91, 92). These pathways are central to endothelial activation and leukocyte recruitment in IgAV, suggesting that S100A10 may amplify local vascular inflammation.

While direct evidence in IgAV is limited, S100A10 likely links innate immunity and vascular injury via plasmin-dependent mechanisms, supporting both acute inflammation and subsequent remodeling.

#### 3.7.2 Clinical relevance of S100A10

Evidence from autoimmune and vascular diseases provides further insight into its potential role. In a study by Salle et al. (n=294) (93), anti-ANXA2 antibodies were found to be significantly more prevalent in patients with systemic autoimmune diseases; however, this difference was not statistically significant within the vasculitis subgroup. In another study by De Angelis et al. (n=3) (94), reduced S100A10 expression was observed in neurons derived from induced pluripotent stem cells obtained from patients with SLE. This finding indicates a possible involvement of S100A10 in the pathogenic mechanisms of systemic autoimmunity.

The expression of the ANXA2–S100A10 complex has also been evaluated in renal pathology. In an immunohistochemical study involving n=25 participants (including patients with LN and n=11 healthy controls), altered expression patterns of ANXA2 and S100A10were observed in diseased tissue. Notably, the expression of these proteins was not static, it varied significantly depending on the severity of vascular lesions. Specifically, alterations in ANXA2 and S100A10 levels correlated with the degree of endothelial injury and vascular damage (95).

#### 3.7.3 Certainty of evidence for S100A10

Certainty of evidence using GRADE assessment for S100A10 was evaluated as very low because key mechanistic insights were exclusively derived from systemic lupus erythematosus models rather than vasculitis. Current evidence in the context of systemic vasculitides is restricted to predictive bioinformatics models without clinical or experimental confirmation.

## 4 Discussion

### 4.1 Summary

Synthesizing the available data, S100 proteins are identified as important components of the vascular inflammatory cascade, playing different roles depending on the specific isoform and stage of the disease. S100A8/9 and S100A12 appear to be the most direct mediators of the acute inflammatory phase. S100A8/9 has demonstrated significant potential as a biomarker for disease activity, supported by moderate certainty of evidence in AAV and KD. While current findings in IgAV are encouraging, they remain limited. Therefore, dedicated prospective studies are essential to confirm the protein’s predictive value and establish its clinical utility for monitoring disease activity in this specific population.

Regarding S100A12, although its role in neutrophil and endothelial activation is established in other vasculitides, no original studies have evaluated it in IgAV patients; thus, prospective clinical validation is essential before it can be considered for disease monitoring.

In contrast, S100A4 and S100A10 appear to link acute inflammation with vascular remodeling and tissue damage. S100A4 mediates pro-inflammatory and profibrotic properties via EMT/EndMT pathways, hypothetically connecting early inflammatory events with later chronic renal damage in IgAV. Meanwhile, S100A10 may contribute to vascular injury by regulating fibrinolysis and promoting endothelial activation. However, evidence for both S100A4 and S100A10 in IgAV remains largely hypothetical and indirect, relying on their known molecular functions rather than comprehensive, disease-specific clinical data. These findings underscore a knowledge gap. Consequently, prospective studies are required to clarify the precise involvement of these S100 proteins in the pathogenesis and monitoring of IgAV.

### 4.2 Conclusion

Collectively, S100 proteins represent potential mediators in the inflammatory cascade of IgAV, though the current evidence remains fragmented. While S100A8/9 shows promise, its clinical utility is limited by inconsistent findings. For S100A12, S100A4, and S100A10, standardized prospective trials are recommended to establish direct clinical evidence before these biomarkers can be validated for risk stratification in clinical practice.

### 4.3 Limitations

Limitations of this review include the variable methodological quality of primary studies (e.g., frequently unreported blinding) and small sample sizes typical of rare diseases. Additionally, substantial heterogeneity in study designs and outcomes precluded quantitative meta-analysis; thus, findings are presented solely as a narrative synthesis. A limitation of this study is the lack of prospective registration in a database such as PROSPERO. While we strictly followed the PRISMA 2020 guidelines to ensure transparency and mitigate reporting bias, the absence of a pre-registered protocol may limit the perceived objectivity of the synthesis.
