# Supplementary material for: S100 proteins in IgA vasculitis and other systemic vasculitides – from pathogenic mechanisms to clinical biomarkers: a systematic review
Source: Front Immunol. 2026 Mar 25;17:1802262. doi: 10.3389/fimmu.2026.1802262 (PMC13056824; doi:10.3389/fimmu.2026.1802262)
Supplement: Supplementary file 2 [file Table2.docx]

Date of search: 18.11.2025

**S100A8/9**

Number of publications after duplicates removal 167; included for full text screening: 59.

| **Database** | **Search strategy** | **Number of publications found** |
| --- | --- | --- |
| PubMed | ("S100A8"[Title/Abstract] OR "S100 A8"[Title/Abstract] OR "S100A9"[Title/Abstract] OR "S100 A9"[Title/Abstract] OR “calprotectin”[Title/Abstract] OR "calgranulin A"[Title/Abstract] OR "calgranulin B"[Title/Abstract] OR “MRP8”[Title/Abstract] OR “MRP14”[Title/Abstract] OR “MRP8/14”[Title/Abstract])  AND  ("IgA vasculitis"[Title/Abstract] OR “IgAV”[Title/Abstract] OR "Henoch-Schönlein purpura"[Title/Abstract] OR "Henoch Schoenlein purpura"[Title/Abstract] OR "HSP"[Title/Abstract] OR "IgA-associated vasculitis"[Title/Abstract] OR "Immunoglobulin A vasculitis"[Title/Abstract] OR "IgA mediated vasculitis"[Title/Abstract] OR "small vessel vasculitis"[Title/Abstract] OR "leukocytoclastic vasculitis"[Title/Abstract] OR “LCV”[Title/Abstract] OR "cryoglobulinemic vasculitis"[Title/Abstract] OR "cryoglobulinemia"[Title/Abstract] OR "immune complex vasculitis"[Title/Abstract] OR "hypersensitivity vasculitis"[Title/Abstract]OR "systemic vasculitis"[Title/Abstract]) | 36 |
| Scopus | "S100A8/9" OR "S100A8" OR "S100 A8" OR "S100A9" OR "S100 A9" OR "calprotectin" OR "calgranulin A" OR "calgranulin B" OR “MRP8” OR “MRP14” OR “MRP8/14“  AND  "IgA vasculitis" OR IgAV OR "Henoch-Schönlein purpura" OR "Henoch Schoenlein purpura" OR HSP OR "IgA-associated vasculitis" OR "Immunoglobulin A vasculitis" OR "IgA mediated vasculitis" OR "small vessel vasculitis" OR "leukocytoclastic vasculitis" OR LCV OR "cryoglobulinemic vasculitis" OR "cryoglobulinemia" OR "immune complex vasculitis" OR "hypersensitivity vasculitis" OR "systemic vasculitis" | 43 |
| Embase | ("S100A8/9":ti,ab OR  "S100A8":ti,ab OR  "S100 A8":ti,ab OR  "S100A9":ti,ab OR  "S100 A9":ti,ab OR  "calprotectin":ti,ab OR  "calgranulin A":ti,ab OR  "calgranulin B":ti,ab OR  “MRP8”:ti,ab OR  “MRP14”:ti,ab OR “MRP8/14”:ti,ab)  AND  ("IgA vasculitis":ti,ab OR IgAV:ti,ab OR "Henoch-Schönlein purpura":ti,ab OR "Henoch Schoenlein purpura":ti,ab OR HSP:ti,ab OR "IgA-associated vasculitis":ti,ab OR "Immunoglobulin A vasculitis":ti,ab OR "IgA mediated vasculitis":ti,ab OR "small vessel vasculitis":ti,ab OR "leukocytoclastic vasculitis":ti,ab OR LCV:ti,ab OR "cryoglobulinemic vasculitis":ti,ab OR "cryoglobulinemia":ti,ab OR "immune complex vasculitis":ti,ab OR "hypersensitivity vasculitis":ti,ab OR "systemic vasculitis":ti,ab) | 69 |
| Web of Science | (TS=("S100A8/9" OR "S100A8" OR "S100 A8" OR "S100A9" OR "S100 A9" OR "calprotectin" OR "calgranulin A" OR "calgranulin B" OR “MRP8” OR “MRP14” OR “MRP8/14”))  AND  (TS=("IgA vasculitis" OR IgAV OR "Henoch-Schönlein purpura" OR "Henoch Schoenlein purpura" OR HSP OR "IgA-associated vasculitis" OR "Immunoglobulin A vasculitis" OR "IgA mediated vasculitis" OR "small vessel vasculitis" OR "leukocytoclastic vasculitis" OR LCV OR "cryoglobulinemic vasculitis" OR "cryoglobulinemia" OR "immune complex vasculitis" OR "hypersensitivity vasculitis" OR "systemic vasculitis")) | 40 |

**S100A12**

Number of publications after duplicates removal 62; included for full text screening: 35.

| **Database** | **Search strategy** | **Number of publications found** |
| --- | --- | --- |
| PubMed | ("S100A12"[Title/Abstract] OR "S100 calcium-binding protein A12"[Title/Abstract] OR "S100-A12"[Title/Abstract] OR "Calgranulin C"[Title/Abstract] OR "Extracellular newly identified RAGE-binding protein"[Title/Abstract] OR "EN-RAGE"[Title/Abstract] OR "p6"[Title/Abstract])  AND  ("IgA vasculitis"[Title/Abstract] OR “IgAV”[Title/Abstract] OR "Henoch-Schönlein purpura"[Title/Abstract] OR "Henoch Schoenlein purpura"[Title/Abstract] OR "HSP"[Title/Abstract] OR "IgA-associated vasculitis"[Title/Abstract] OR "Immunoglobulin A vasculitis"[Title/Abstract] OR "IgA mediated vasculitis"[Title/Abstract] OR "small vessel vasculitis"[Title/Abstract] OR "leukocytoclastic vasculitis"[Title/Abstract] OR “LCV”[Title/Abstract] OR "cryoglobulinemic vasculitis"[Title/Abstract] OR "cryoglobulinemia"[Title/Abstract] OR "immune complex vasculitis"[Title/Abstract] OR  "hypersensitivity vasculitis"[Title/Abstract]OR "systemic vasculitis"[Title/Abstract]) | 16 |
| Scopus | "S100A12" OR  "S100 calcium-binding protein A12" OR  "S100-A12" OR  "Calgranulin C" OR  "Extracellular newly identified RAGE-binding protein" OR  "EN-RAGE" OR  "p6"  AND  "IgA vasculitis" OR IgAV OR "Henoch-Schönlein purpura" OR "Henoch Schoenlein purpura" OR HSP OR "IgA-associated vasculitis" OR "Immunoglobulin A vasculitis" OR "IgA mediated vasculitis" OR "small vessel vasculitis" OR "leukocytoclastic vasculitis" OR LCV OR "cryoglobulinemic vasculitis" OR "cryoglobulinemia" OR "immune complex vasculitis" OR "hypersensitivity vasculitis" OR "systemic vasculitis" | 19 |
| Embase | ("S100A12":ti,ab OR "S100 calcium-binding protein A12":ti,ab OR "S100-A12":ti,ab OR "Calgranulin C":ti,ab OR "Extracellular newly identified RAGE-binding protein":ti,ab OR "EN-RAGE":ti,ab OR "p6":ti,ab) AND ("IgA vasculitis":ti,ab OR IgAV:ti,ab OR "Henoch-Schönlein purpura":ti,ab OR "Henoch Schoenlein purpura":ti,ab OR HSP:ti,ab OR "IgA-associated vasculitis":ti,ab OR "Immunoglobulin A vasculitis":ti,ab OR "IgA mediated vasculitis":ti,ab OR "small vessel vasculitis":ti,ab OR "leukocytoclastic vasculitis":ti,ab OR LCV:ti,ab OR "cryoglobulinemic vasculitis":ti,ab OR "cryoglobulinemia":ti,ab OR "immune complex vasculitis":ti,ab OR "hypersensitivity vasculitis":ti,ab OR "systemic vasculitis":ti,ab) | 21 |
| Web of Science | (TS=("S100A12" OR  "S100 calcium-binding protein A12" OR  "S100-A12" OR  "Calgranulin C" OR  "Extracellular newly identified RAGE-binding protein" OR  "EN-RAGE" OR  "p6"))  AND  (TS=("IgA vasculitis" OR IgAV OR "Henoch-Schönlein purpura" OR "Henoch Schoenlein purpura" OR HSP OR "IgA-associated vasculitis" OR "Immunoglobulin A vasculitis" OR "IgA mediated vasculitis" OR "small vessel vasculitis" OR "leukocytoclastic vasculitis" OR LCV OR "cryoglobulinemic vasculitis" OR "cryoglobulinemia" OR "immune complex vasculitis" OR "hypersensitivity vasculitis" OR "systemic vasculitis")) | 19 |

**S100A4**

Number of publications after duplicates removal 20; included for full text screening: 4.

| **Database** | **search strategy** | **Number of publications found** |
| --- | --- | --- |
| PubMed | ("S100A4"[Title/Abstract]  OR "S100-A4"[Title/Abstract]  OR "metastasin"[Title/Abstract]  OR "S100 calcium-binding protein A4"[Title/Abstract]  OR "fibroblast specific protein 1"[Title/Abstract]  OR "FSP1"[Title/Abstract]  OR "Calvasculin"[Title/Abstract]  OR "MTS1"[Title/Abstract]  OR "pEL98"[Title/Abstract])  AND  ("IgA vasculitis"[Title/Abstract] OR “IgAV”[Title/Abstract] OR "Henoch-Schönlein purpura"[Title/Abstract] OR "Henoch Schoenlein purpura"[Title/Abstract] OR "HSP"[Title/Abstract] OR "IgA-associated vasculitis"[Title/Abstract] OR "Immunoglobulin A vasculitis"[Title/Abstract] OR "IgA mediated vasculitis"[Title/Abstract] OR "small vessel vasculitis"[Title/Abstract] OR "leukocytoclastic vasculitis"[Title/Abstract] OR “LCV”[Title/Abstract] OR "cryoglobulinemic vasculitis"[Title/Abstract] OR "cryoglobulinemia"[Title/Abstract] OR "immune complex vasculitis"[Title/Abstract] OR  "hypersensitivity vasculitis"[Title/Abstract]OR "systemic vasculitis"[Title/Abstract]) | 6 |
| Scopus | "S100A4" OR  "S100-A4" OR  "S100A4 protein" OR  "metastasin" OR  "MTS1" OR  "S100 calcium-binding protein A4" OR  "FSP1" OR  "fibroblast specific protein 1" OR  "Calvasculin" OR  "pEL98"  AND  "IgA vasculitis" OR IgAV OR "Henoch-Schönlein purpura" OR "Henoch Schoenlein purpura" OR HSP OR "IgA-associated vasculitis" OR "Immunoglobulin A vasculitis" OR "IgA mediated vasculitis" OR "small vessel vasculitis" OR "leukocytoclastic vasculitis" OR LCV OR "cryoglobulinemic vasculitis" OR "cryoglobulinemia" OR "immune complex vasculitis" OR "hypersensitivity vasculitis" OR "systemic vasculitis" | 11 |
| Embase | ("S100A4":ti,ab OR "S100-A4":ti,ab OR "S100A4 protein":ti,ab OR "metastasin":ti,ab OR "MTS1":ti,ab OR "S100 calcium-binding protein A4":ti,ab OR "FSP1":ti,ab OR "fibroblast specific protein 1":ti,ab OR "Calvasculin":ti,ab OR "pEL98":ti,ab) AND ("IgA vasculitis":ti,ab OR IgAV:ti,ab OR "Henoch-Schönlein purpura":ti,ab OR "Henoch Schoenlein purpura":ti,ab OR HSP:ti,ab OR "IgA-associated vasculitis":ti,ab OR "Immunoglobulin A vasculitis":ti,ab OR "IgA mediated vasculitis":ti,ab OR "small vessel vasculitis":ti,ab OR "leukocytoclastic vasculitis":ti,ab OR LCV:ti,ab OR "cryoglobulinemic vasculitis":ti,ab OR "cryoglobulinemia":ti,ab OR "immune complex vasculitis":ti,ab OR "hypersensitivity vasculitis":ti,ab OR "systemic vasculitis":ti,ab) | 10 |
| Web of Science | (TS=("S100A4" OR  "S100-A4" OR  "S100A4 protein" OR  "metastasin" OR  "MTS1" OR  "S100 calcium-binding protein A4" OR  "FSP1" OR  "fibroblast specific protein 1" OR  "Calvasculin" OR  "pEL98"))  AND  (TS=("IgA vasculitis" OR IgAV OR "Henoch-Schönlein purpura" OR "Henoch Schoenlein purpura" OR HSP OR "IgA-associated vasculitis" OR "Immunoglobulin A vasculitis" OR "IgA mediated vasculitis" OR "small vessel vasculitis" OR "leukocytoclastic vasculitis" OR LCV OR "cryoglobulinemic vasculitis" OR "cryoglobulinemia" OR "immune complex vasculitis" OR "hypersensitivity vasculitis" OR "systemic vasculitis")) | 8 |

**S100A10**

Number of publications after duplicates removal 3; included for full text screening: 0.

| **Database** | **Search strategy** | **Number of publications found** |
| --- | --- | --- |
| PubMed | ("S100A10"[Title/Abstract] OR "S100 calcium-binding protein A10"[Title/Abstract] OR "S100-A10"[Title/Abstract] OR "annexin II light chain"[Title/Abstract] OR "ANX2L"[Title/Abstract] OR "ANX2LG"[Title/Abstract] OR "CAL1L"[Title/Abstract] OR "CLP11"[Title/Abstract])  AND  ("IgA vasculitis"[Title/Abstract] OR “IgAV”[Title/Abstract] OR "Henoch-Schönlein purpura"[Title/Abstract] OR "Henoch Schoenlein purpura"[Title/Abstract] OR "HSP"[Title/Abstract] OR "IgA-associated vasculitis"[Title/Abstract] OR "Immunoglobulin A vasculitis"[Title/Abstract] OR "IgA mediated vasculitis"[Title/Abstract] OR "small vessel vasculitis"[Title/Abstract] OR "leukocytoclastic vasculitis"[Title/Abstract] OR “LCV”[Title/Abstract] OR "cryoglobulinemic vasculitis"[Title/Abstract] OR "cryoglobulinemia"[Title/Abstract] OR "immune complex vasculitis"[Title/Abstract] OR  "hypersensitivity vasculitis"[Title/Abstract]OR "systemic vasculitis"[Title/Abstract]) | 2 |
| Scopus | "S100A10" OR "S100 calcium-binding protein A10" OR "S100-A10" OR "annexin II light chain" OR "ANX2L" OR "ANX2LG" OR "CAL1L" OR "CLP11"  AND  "IgA vasculitis" OR IgAV OR "Henoch-Schönlein purpura" OR "Henoch Schoenlein purpura" OR HSP OR "IgA-associated vasculitis" OR "Immunoglobulin A vasculitis" OR "IgA mediated vasculitis" OR "small vessel vasculitis" OR "leukocytoclastic vasculitis" OR LCV OR "cryoglobulinemic vasculitis" OR "cryoglobulinemia" OR "immune complex vasculitis" OR "hypersensitivity vasculitis" OR "systemic vasculitis" | 3 |
| Embase | ("S100A10":ti,ab OR "S100 calcium-binding protein A10":ti,ab OR "S100-A10":ti,ab OR "annexin II light chain":ti,ab OR "ANX2L":ti,ab OR "ANX2LG":ti,ab OR "CAL1L":ti,ab OR "CLP11":ti,ab ) AND ("IgA vasculitis":ti,ab OR IgAV:ti,ab OR "Henoch-Schönlein purpura":ti,ab OR "Henoch Schoenlein purpura":ti,ab OR HSP:ti,ab OR "IgA-associated vasculitis":ti,ab OR "Immunoglobulin A vasculitis":ti,ab OR "IgA mediated vasculitis":ti,ab OR "small vessel vasculitis":ti,ab OR "leukocytoclastic vasculitis":ti,ab OR LCV:ti,ab OR "cryoglobulinemic vasculitis":ti,ab OR "cryoglobulinemia":ti,ab OR "immune complex vasculitis":ti,ab OR "hypersensitivity vasculitis":ti,ab OR "systemic vasculitis":ti,ab) | 3 |
| Web of Science | (TS=("S100A10" OR "S100 calcium-binding protein A10" OR "S100-A10" OR "annexin II light chain" OR "ANX2L" OR "ANX2LG" OR "CAL1L" OR "CLP11"))  AND  (TS=("IgA vasculitis" OR IgAV OR "Henoch-Schönlein purpura" OR "Henoch Schoenlein purpura" OR HSP OR "IgA-associated vasculitis" OR "Immunoglobulin A vasculitis" OR "IgA mediated vasculitis" OR "small vessel vasculitis" OR "leukocytoclastic vasculitis" OR LCV OR "cryoglobulinemic vasculitis" OR "cryoglobulinemia" OR "immune complex vasculitis" OR "hypersensitivity vasculitis" OR "systemic vasculitis")) | 2 |
